# Microbiome Resilience and Health Implications for People in Half-Year Travel

**DOI:** 10.3389/fimmu.2022.848994

**Published:** 2022-02-24

**Authors:** Mingyue Cheng, Hong Liu, Maozhen Han, Shuai Cheng Li, Dongbo Bu, Shiwei Sun, Zhiqiang Hu, Pengshuo Yang, Rui Wang, Yawen Liu, Feng Chen, Jianjun Peng, Hong Peng, Hongxing Song, Yang Xia, Liqun Chu, Quan Zhou, Feng Guan, Jing Wu, Guangming Tan, Kang Ning

**Affiliations:** ^1^Key Laboratory of Molecular Biophysics of the Ministry of Education, Hubei Key Laboratory of Bioinformatics and Molecular-Imaging, Center of AI Biology, Department of Bioinformatics and Systems Biology, College of Life Science and Technology, Huazhong University of Science and Technology, Wuhan, China; ^2^Beijing Shijitan Hospital, Capital Medical University, Beijing, China; ^3^Department of Computer Science, City University of Hong Kong, Hong Kong, Hong Kong SAR, China; ^4^Key Lab of Intelligent Information Processing, State Lab of Computer Architecture, Institute of Computing Technology, Chinese Academy of Sciences, Beijing, China; ^5^School of Computer and Control, University of Chinese Academy of Sciences, Beijing, China

**Keywords:** travel, enterotype, microbiome, bi-directional plasticity, resilience, dietary shift, immunity, health

## Abstract

Travel entail change in geography and diet, both of which are known as determinant factors in shaping the human gut microbiome. Additionally, altered gut microbiome modulates immunity, bringing about health implications in humans. To explore the effects of the mid-term travel on the gut microbiome, we generated 16S rRNA gene and metagenomic sequencing data from longitudinal samples collected over six months. We monitored dynamic trajectories of the gut microbiome variation of a Chinese volunteer team (VT) in their whole journey to Trinidad and Tobago (TAT). We found gut microbiome resilience that VT’s gut microbial compositions gradually transformed to the local TAT’s enterotypes during their six-month stay in TAT, and then reverted to their original enterotypes after VT’s return to Beijing in one month. Moreover, we identified driven species in this bi-directional plasticity that could play a role in immunity modulation, as exemplified by *Bacteroides dorei* that attenuated atherosclerotic lesion formation and effectively suppressed proinflammatory immune response. Another driven species *P. copri* could play a crucial role in rheumatoid arthritis pathogenesis, a chronic autoimmune disease. Carbohydrate-active enzymes are often implicated in immune and host-pathogen interactions, of which glycoside hydrolases were found decreased but glycosyltransferases and carbohydrate esterases increased during the travel; these functions were then restored after VT’ returning to Beijing. Furthermore, we discovered these microbial changes and restoration were mediated by VT people’s dietary changes. These findings indicate that half-year travel leads to change in enterotype and functional patterns, exerting effects on human health. Microbial intervention by dietary guidance in half-year travel would be conducive to immunity modulation for maintaining health.

## Introduction

Human gut microbes form dynamic and interweaved communities (1, 2), shaped by environmental factors such as geography and diet (3, 4). Previous studies have indicated that the human gut microbiome can respond rapidly to short-term environmental changes (5–7). In comparison, during long-term (> 1 year) environmental changes, the composition of an individual’s gut microbial community is predominantly determined by dietary habits (8, 9); such dynamics of the gut microbial community are highly variable among individuals (10, 11).

Immigration brings about long-term changes in geography and diet, leading to variation of the human gut microbiome (12). Vangay et al. investigated the dynamics of the gut microbiome during migration from a non-Western country to the United States of America (USA), demonstrating a westernization of an immigrated individual’s gut microbiome whose diversity was greatly decreased and that the USA-associated strains became dominated in the gut microbiome (12). Moreover, a study conducted on Irish Traveller revealed that the gut microbiome of Irish Traveler has gradually shifted from the non-industrialized pattern to an industrialized pattern, correlated with the degree to which Travellers have adopted the new non-nomadic lifestyle (13). Furthermore, the westernized or industrialized gut microbiome might increase the risk of obesity (12), and the risk of auto-immune disorders and chronic diseases *via* increasing in the generation of secondary bile acids, LPS biosynthesis, and the ratio of trimethylamine-producing to trimethylamine-consuming bacteria (13).

Traveling abroad, as one of the common activities, entails changes in geography and diet for days or months, whose influences in gut microbiome remain poorly understood. Our previous study investigated gut microbial communities of a Chinese volunteer team (VT) consisting of 10 people who departed from Beijing, stayed for six months in Trinidad and Tobago (TAT) and returned to Beijing. We found the gut microbial communities of VT members switched to the patterns of gut microbial communities of TAT people during their time in TAT and restored to their original patterns after they returned to Beijing in one month (14). However, the 16S rRNA gene sequencing limited the detection of the microbiome at the species and functional level, and their potential health implications for people during this six-month travel.

In this study, we conducted a high-density longitudinal sampling and integrated 16S rRNA gene and metagenomic sequencing data, and dietary records to depict the microbiome variation of VT people in their whole half-year travel to TAT. We tracked the dynamic trajectories of gut microbiome variation in the form of enterotypes. We recognized the driven microbial species and functional changes beneath the switching and restoring of enterotypes in the travel, which were mediated by the change and restoring of the diet. Furthermore, the health implications resulted from the microbiome variation warranted dietary guidance for microbial intervention during half-year travel.

## Materials and Methods

### Collection of Human Fecal Samples and Dietary Information

Fecal samples were collected from each individual of the Chinese VT with a high sampling frequency (**Supplementary Figure S1A**). The collection locations of these samples included Beijing and TAT. Accordingly, these samples were subdivided into three groups, including the Chinese VT (10 individuals) that stayed in Beijing before leaving for TAT (VTC, 15–22 December 2015, 20 samples), the Chinese VT that stayed in TAT (VTT, from 31 December 2015 to June 2016, 109 samples), and the Chinese VT that returned to Beijing after a long stay (VTB, July 2016, 55 samples). These samples were grouped into six phases (**Supplementary Figure S1A**) along the time series, including T1 (20 samples), T2 (28 samples), T3 (60 samples), T4 (21 samples), T5 (35 samples), and T6 (20 samples). T1 represented the pre-travel time; T2, T3, and T4 represented three times during the stay of the VT in TAT; and T5 and T6 represented two times after the VT returned to Beijing, China. T1 belonged to the VTC group; T2, T3, and T4 belonged to the VTT group; and T5 and T6 belonged to the VTB group.

Fecal samples from TTNs (14 individuals, 28 samples; **Supplementary Figure S1B**), TTPs (3 individuals, 6 samples; **Supplementary Figure S1C**), TTCs staying in TAT for more than 1 year (4 individuals, 8 samples; **Supplementary Figure S1C**), and BJNs (10 individuals, 57 samples; **Supplementary Figure S1D**) were used as controls. These fecal samples were kept at -20°C for less than 1 week before transfer to the laboratory of the Beijing Genomics Institute and then stored at -80°C until DNA extraction. Dietary information was collected and recorded for each individual during the long stay.

### DNA Extraction and Sequencing

DNA extraction from fecal samples was performed using a PowerSoil DNA Isolation Kit (MoBio, USA) following the manufacturer’s instructions. Extracted DNA was dissolved in TE buffer and stored at -20°C until further use. To characterize the taxonomic profile of the gut microbial community, the V4 hypervariable region of the microbial 16S rRNA gene was amplified using the universal bacterial/archaeal primers 515F (5′-GTGCCAGCMGCCGCGGTAA-3′) and 806R (5′-GGACTACHVGGGTWTCTAAT-3′). Fusion primers with dual indexes and adapters were used for a polymerase chain reaction, and the jagged ends of the DNA fragments were converted into blunt ends using T4 DNA polymerase, Klenow fragment, and T4 Polynucleotide Kinase. Then, an ‘A’ base was appended to each 3′ end to facilitate the addition of adapters. Next, short fragments were removed using Ampure beads. Finally, the qualified libraries were used for sequencing on the Illumina MiSeq platform using paired-end sequencing technology (2 × 250 bp).

To characterize gut microbiome functional profiles, 62 fecal samples were selected for shotgun metagenome sequencing (**Supplementary Figure S1**), including the fecal samples of three individuals: VT3, VT6, and VT10. Metagenomic DNA of samples was extracted, fragmented randomly to the desired size using a Covaris S/E210 or Bioruptor, and electrophoresed to yield the required lengths of DNA fragments. Subsequently, adapters were ligated to DNA fragments, and these fragments were evaluated for cluster preparation. Sequencing was performed with an insert size of 350 bp on an Illumina Xten platform.

### 16S rRNA Gene Sequencing Data Processing

All 16S rRNA raw data were preprocessed to obtain clean data, and two paired-end reads were generated using Fast Length Adjustment of Short reads (v1.2.11) (15). Specifically, the threshold of the minimal overlapping length was set to 15 bp, and the mismatch ratio of the overlapped region was no more than 0.1. High-quality paired-end reads were combined into tags based on the overlaps. Putative chimeras were identified using the SILVA database (16) (Release 123) and removed using the ‘chimera.uchime’ and ‘remove.seqs’ commands in Mothur (17). Existing tools for analyzing the microbial community include Quantitative Insights Into Microbial Ecology (QIIME) (18), DADA2 (19), and Deblur (20). In this study, QIIME was applied to analyze the 16S amplicon data. All high-quality sequences of human fecal samples (287 samples) were aligned using PyNAST (21) and dereplicated using UCLUST in QIIME (v1.9.1) (18). Finally, the Greengenes database (22) (version 13_8) was applied as the reference database for OTU classification of *de novo* OTUs that were clustered at 97% nucleotide identity threshold. To remove singleton OTUs, the minimum reads per OTU threshold was set to 2. The returned reads per sample varied from 51,837 to 335,665 (average = 124,944 reads per sample). The resulting OTU table containing 287 samples was rarefied to 51,837 reads to remove biases from variations in sample read numbers.

### Microbial Diversity Assessment

Microbial alpha- and beta-diversity values were determined using the QIIME (18) pipeline. For alpha-diversity, rarefaction curves were drawn based on the richness metrics, observed OTUs, Chao1 index, the Shannon evenness metric, and the Simpson evenness metric. For beta-diversity analysis, the final OTU table was rarefied to contain 51,837 reads per sample. Pearson correlation, Spearman correlation, and weighted UniFrac distance metrics (23) were adopted to measure community similarity between samples. Microbial community clustering was arrayed by PCoA, visualized using ggplot packages in R, and modified with Adobe Illustrator.

### Analysis of Microbial CAGs

The top 109 genera were selected based on the average abundance of the genus, and the associations between individual genera were determined using Kendall’s correlation coefficient. The full set of associations was calculated in R with the ‘cor’ package. The function *pheatmap* in the ‘pheatmap’ package was used to visualize and cluster these associations in R, whereby the hierarchical clustering was grouped based on the Spearman correlation coefficient, and Ward clustering was applied to capture CAGs at the genus level. All *p* values of these associations were corrected for multiple testing using the Benjamini and Hochberg false discovery rate (FDR)-controlling procedure (24). The cutoff of the FDR-corrected p-value was set at 0.05, and significant associations were imported to Cytoscape (25), which was employed to visualize the resulting networks. The nodes (genera) were grouped based on the results of clustering in pheatmap.

To evaluate the dynamic process of the microbial community, to quantitatively measure the plasticity of the gut microbiome, we proposed the adaptation index. The index was calculated based on **Equation (1)**.


(1) $${\text{D}}_{1,2}=\sqrt[{2}]{\Sigma_{i=1}^{n}{\left(x_{1i}-x_{2i}\right)}^{2}/n}$$

In this equation, D_1, 2_ is the distance between control (TTN) and the Chinese volunteer team in the different phases (or groups) based on the compositional data of the microbial community. Specifically, *x_1_* represents the average abundance of the certain genus in top n (109 in this study) genera of the gut microbial community for the volunteer team in one specific phase (out of T1 to T6), and *x_2_* represents the average abundance of certain genus in top n (109 in this study) genera of the gut microbial community of TTN.

### Enterotype Analysis

The enterotype of each fecal sample was analyzed using the PAM method, which analyzes the Jensen-Shannon (JS) distance among samples based on the relative abundance of genera in each community (26). Specifically, before calculating, more abundant genera were selected by setting the threshold of average relative abundance to 10^-4^ (27). JS distances among samples based on the selected genus-level relative abundance were calculated. CH indexes, as previously described, were applied to choose the optimal number of clusters (27, 28).

### Functional Profiling Based on the 16S rRNA Datasets

In this study, 62 fecal samples were selected for shotgun metagenomic sequencing. For association studies, we also performed functional predictions for all samples with coarse granularity. We used PICRUSt (29) (version 1.0.0-dev) to make functional predictions based on the 16S rDNA dataset from each sample. In this work, PICRUSt was applied to predict the functional composition of each fecal sample according to the manufacturer’s instructions. Specifically, the ‘pick_closed_reference_otus.py’ command in QIIME was performed on all quality-filtered sequence data to pick OTUs. For clustering the OTUs, 97% nucleotide identity against the Greengenes database (22) (version 13_8) was set as the threshold. The OTU table was normalized using the ‘normalize_by_copy_number.py’ command. The normalized OTU table was used for functional prediction with the ‘predict_metagenomes.py’ script, and functional trait abundances were determined for each sample using the KEGG database (30) (version 66.1, May 1, 2013). Finally, the predicted functional content collapsed to level three of the KEGG hierarchy using the ‘categorize_by_function.py’ script.

### Analysis of the Associations Between Diet and Microbial Community Composition

The associations between diet and microbial community composition were calculated based on (i) compositional data, which includes taxonomic composition (relative taxonomic abundances) and functional composition at KEGG module-level three; (ii) dietary information. To preprocess compositional data, the original relative abundance [plus a very small value (1E-20) as suggested by (31)] for each OTU was filtered, and log-transformation was then applied to generate the relative abundances. Similarly, for dietary information, the values of each variable were transformed to *z*-scores. Based on Euclidean distances, the Mantel correlations between compositional data and dietary information (9,999 permutations) were calculated, and the results were obtained in R (version 3.3.1) and visualized in Adobe Illustrator (version 16.0.0). Correlations between taxonomic composition data and functional composition data were determined for each diet using Mantel’s tests (31).

### Metagenomic Sequence Data Processing

In this study, 62 fecal samples (**Supplementary Figure S1**), obtained from VT3, VT6, and VT10, were selected for metagenomic sequencing. The generated reads were quality filtered and trimmed by removing reads containing 10% or more ambiguous bases (N base), adapter sequences, and 50% or more low-quality (Q < 20) bases. In addition, reads that could be perfectly aligned to the human genome were removed. Finally, 53.23 Gb of high-quality sequences on average for each sample (fastq document) was acquired, producing a total of 3.3 TB of sequence data (fastq document; **Supplementary Table 1**).

### Genome Assembly

*De novo* metagenome assembly was performed for 62 metagenome datasets using MEGAHIT v1.1.1-2-g02102e1 (32), with option –meta-large and with a *k*-mer list of 27, 37, 47, 57, 67, 77, 87, 97, 107, 117, and 127. Contigs larger than 500 bp were kept for further analysis. These contigs exhibited an average N50 length of 6,303 bp and ranged from 910 to 10,685 bp (**Supplementary Table 1**).

### Non-Redundant Gene Catalogue Construction

To predict microbial genes and proteins for each of the 62 fecal samples, Prodigal v2.6 (33) in “Meta” mode was applied to recognize open reading frames (ORFs) and proteins in assembled contigs. The program reported an average of 499,729 proteins for each sample (ranging from 207,485 to 1,049,111; **Supplementary Table 1**). Among these proteins, 358,116 were complete proteins for each sample, whereas 141,613 proteins were incomplete (**Supplementary Table 1**). These complete proteins were selected as the nonredundant protein set for each sample, which was built by pairwise comparisons of all predicted proteins of each sample using CD-HIT (34). The redundant proteins were removed using the following criteria: 90% identity over 90% of the short protein length and over 90% of the long protein length. On average, 333,881 unique proteins (ranging from 143,391 to 677,756) for these samples were obtained.

### Gene Functional Classification and Ortholog Group Profiling

To identify CAZymes in each sample, CAZyme screening of these nonredundant proteins was performed. According to the manual of dbCAN CAZyme annotation, all completed and putative proteins were annotated by searching against entries in the local CAZy database, which was downloaded from dbCAN (35) (http://csbl.bmb.uga.edu/dbCAN/). The proportion of each component of CAZyme in each sample was computed by dividing the number of each component of the CAZyme by the total number of identified CAZyme components in that sample. Based on the components of CAZyme in each sample, PCA was applied to show the trajectory of each sample, which was colored according to the group information. By using Euclidean distances based on samples’ functions, PERMANOVA tests with 9,999 permutations were applied to compare differences among samples grouped by enterotype. Additionally, linear discriminant analysis was used to utilize a linear combination of CAZyme features (top 19 components of CAZyme) to maximize the separation of the groups. Furthermore, based on the top 19 components of CAZyme, heatmap analysis was performed to illustrate the clustering results of CAZyme features and the discernibility of these CAZyme features in different groups.

### Shotgun Metagenomics Analysis for Species Composition and Functional Composition

Shotgun metagenomics sequence data of 62 fecal samples were identified at the species level *via* MetaPhlAn2 with default settings (36). Taxonomical information at the species level for these 62 fecal samples was obtained. Functional annotations were identified using the HuMAnN2 (37) pipeline with UniRef50 (38). The functional pathways were annotated by mapping reads to MetaCyc databases (39). Significantly enriched pathways were identified among enterotypes 1 and 2 compared with enterotype 3 with 2-fold changes.

### Data Accession

All sequencing data (including 287 16S rRNA data and 62 metagenomic data) for fecal samples were deposited in NCBI’s Sequence Read Archive database under Bioproject number PRJNA393237 and can also be viewed in NODE (http://www.biosino.org/node) by pasting the accession (OEP000187) into the text search box or at the URL http://www.biosino.org/node/project/detail/OEP000187.

## Results

### Study Design and Population

In this work, we collected 287 fecal samples from 41 individuals, including a Chinese VT (10 individuals, 188 samples), Beijing healthy natives (BJNs, 10 individuals, 57 samples), TAT healthy natives (TTNs, 14 individuals, 28 samples), TAT patients (TTPs, 3 individuals, 6 samples), and TAT Chinese individuals (TTCs, 4 individuals, 8 samples), reflecting high-density longitudinal sampling. We followed individuals for their entire journeys of more than 8 months (including 1 month before and 1 month after travel) and partitioned their journeys into six phases (T1–T6). Specifically, T1 represented the pretravel time; T2, T3, and T4 represented three times during the stay of the VT in TAT; and T5 and T6 represented two times after the VTs returned to Beijing (**Supplementary Figure S1**). Finally, we sequenced the V4 hypervariable region of the microbial 16S rRNA genes and clustered these fecal samples into enterotypes to investigate the relationships between resilience and enterotype. We sequenced 62 of the samples, mostly from VT3, VT6, and VT10, to obtain shotgun metagenomics data for exploring strain-level variations and metabolic differences.

### Enterotype-Dependent Resilience Pattern of Taxonomical Structure for Gut Microbial Communities

**Figure 1:**
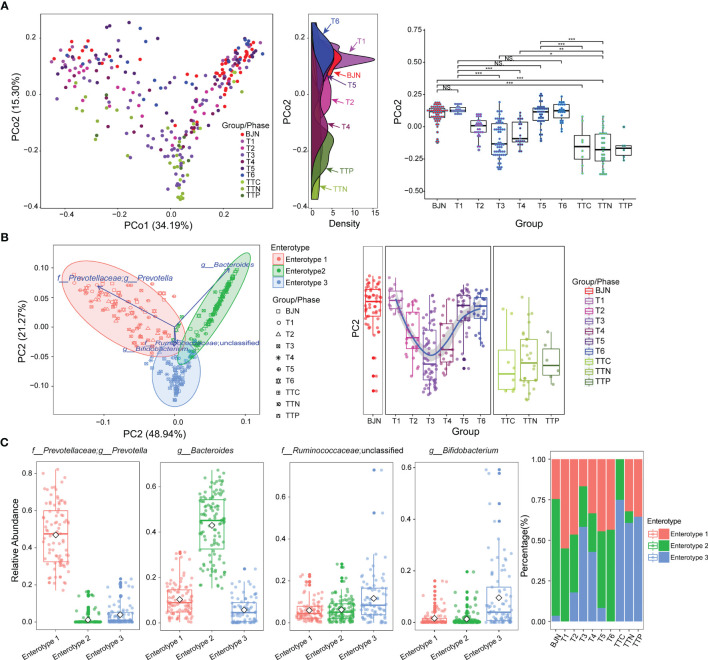
The resilience of human gut microbial communities was bidirectional and was associated with enterotype during the long stay. **(A)** The microbial community compositions of the volunteer team individuals during the study (T1–T6 phases) and those of the control group (BJNs, TTCs, TTNs, and TTPs) plotted in a weighted UniFrac PCoA (left panel). The density curve, which was plotted based on the PCo2 value of each group, showed the distributions of each group of the human gut microbial community (middle panel). The boxplots showed sample distributions against PCo2 of each group (right panel). Statistical significance is tested using Wilcoxon test, ***P < 0.001, **P < 0.01, *P < 0.05, and NS., not significant. **(B)** In total, 287 samples were clustered into three enterotypes based on PCA at the genus level. The majority of samples collected after the VT members returned to Beijing and before departure belonged to clusters of enterotype 1 (pink) and enterotype 2 (green). Together with control fecal samples collected from hosts in Trinidad and Tobago (TTCs, TTNs, and TTPs), the majority of fecal samples collected from TAT (T2–T4) belonged to the cluster of enterotype 3 (blue). **(C)** The major contributors in these three enterotypes were *Prevotella*, *Bacteroides*, Ruminococcaceae_unclassified, and *Bifidobacterium*. **(D)** The distribution of three enterotypes in each group/phase during the stay.

Microbial communities of the samples collected in Beijing before and after the long stays were indistinguishable (*P* = 0.18 and *P* = 0.71, Wilcoxon test; **Figure 1A**). However, samples collected in Beijing were both distinguishable from their respective samples collected during their stay in TAT (*P* = 5.5 × 10^-10^ and *P* = 2.7 × 10^-10^, Wilcoxon test; **Figure 1A**).

We also found that the gut microbial communities of VT1, VT2, VT3, VT4, VT6, and VT8 formed one cluster, and the gut microbial communities of VT5, VT7, VT9, and VT10 formed another cluster before they departed from Beijing. The gut microbial communities of VT members gradually transformed to those similar to the natives during their stay in TAT and then reverted to their respective original community structures after returning to Beijing. The dynamic trajectories of VT1, VT2, VT3, VT4, VT6, and VT8 could be regarded as path 1, whereas those of VT5, VT7, VT9, and VT10 could be regarded as path 2. The results revealed that the dynamic changes of resilience had two paths across the PCoA axis for VT members (**Figure 1A**).

To investigate the characteristics of resilience, we performed enterotype analysis on 287 fecal samples and clustered these samples into three enterotypes (**Figure 1B**). The dominant genera in enterotype 1 and enterotype 2 were *Prevotella* and *Bacteroides*, respectively, whereas the enriched genera in enterotype 3 were Ruminococcaceae_unclassified and *Bifidobacterium* (**Figure 1C**). We observed the bidirectional plastic pattern and resilience in the enterotypes of VT members. Specifically, the results of the enterotype analysis revealed that the fecal samples of most of BJNs and all VT members at the T1 phase exhibited two different gut microbial communities, i.e., enterotype 1 and enterotype 2 (26), whereas the fecal samples of most of TTNs belonged to enterotype 3 (**Figure 1D**). During the long stay at TAT, most samples from VT members had enterotypes that evolved towards that of the TAT natives, despite significant differences among their original enterotypes (**Figures 1B, D**). Notably, their enterotypes quickly returned to their respective original enterotypes after the VT members returned to Beijing (**Figures 1B, D**). The results revealed that the bidirectional resilience of human gut microbial communities was specific to enterotype. Based on the grouping strategy in this study, we observed that these alterations in gut microbial communities were triggered immediately after airplane travel, lasting for 1 month, after which the taxonomical structures of microbial communities resemble those of the natives and showed significant stability.

### Microbial Drivers at the Genus Level for the Enterotype-Dependent Resilience of Human Microbial Communities

**Figure 2:**
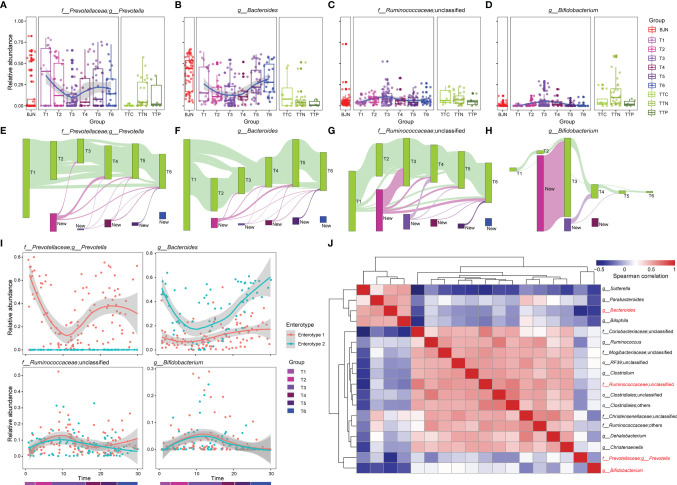
The dynamics of four dominant genera and the bidirectional resilience of the gut microbial community were associated with enterotype. **(A–D)** Dynamics of the average relative abundances of *Prevotella*, *Bacteroides*, Ruminococcaceae_unclassified, and *Bifidobacterium* along the temporal axis. OTUs that were shared by at least 10% of fecal samples during the same phase (green bars) were tracked using Sankey plots for **(E)** *Prevotella*, **(F)** *Bacteroides*, **(G)** Ruminococcaceae_unclassified, and **(H)** *Bifidobacterium*. The heights of bars are proportional to the number of OTUs, and phases are arranged in chronological order. The newly introduced OTUs of each phase are differently colored. The lines represent the transfer of OTUs between phases and are colored by the first phase of appearance. **(I)** Dynamic changes in *Prevotella*, *Bacteroides*, Ruminococcaceae_unclassified, and *Bifidobacterium* for VT members along the temporal axis. **(J)** Co-occurrence patterns between the four dominant genera with other genera across the 287 samples, as determined by Spearman’s rank correlation analysis.

To elucidate the drivers of bidirectional resilience of human gut communities for hosts with different enterotypes, we compared dynamic changes in four representative genera in enterotypes, including their relative abundances (**Figures 2A–D**) and the operational taxonomic units (OTUs) that were maintained in these genera (**Figures 2E–H**). We found that the taxonomical structures of the gut microbial community shifted considerably during the stay. The relative abundances of *Prevotella*, *Bacteroides*, Ruminococcaceae_unclassified, and *Bifidobacterium* showed dramatic changes. The average relative abundances of *Prevotella* and *Bacteroides* decreased when VT members reached TAT and increased when VT members returned to Beijing (**Figures 2A, B**), whereas the average relative abundances of Ruminococcaceae_unclassified and *Bifidobacterium* first increased and then decreased (**Figures 2C, D**). Moreover, by tracing and comparing common OTUs that were present in at least 10% of VT members, we found that individual OTUs within *Prevotella*, *Bacteroides*, Ruminococcaceae_unclassified, and *Bifidobacterium* exhibited distinguished temporal dynamics and showed a plastic pattern during the long stay (**Figures 2E–H**). Many of the OTUs belonging to *Prevotella* displayed plasticity; 71.73% of the OTUs disappeared between the T3 phase and T1 phase, and 39.55% recurred after the VT members returned to Beijing (**Figure 2E**). In contrast, 72.18% of *Bacteroides* OTUs disappeared after arrival to TAT, and 47.75% of *Bacteroides* OTUs recurred after return to Beijing (**Figure 2F**). This plastic pattern was also observable for certain OTUs in Ruminococcaceae_unclassified and *Bifidobacterium* (**Figures 2G, H**). Many Ruminococcaceae_unclassified OTUs (1,101, 56.17%) and *Bifidobacterium* OTUs (79, 75.24%) appeared at the T2 phase and a large proportion of these OTUs (43.05% and 79.75%) still existed in the T3 phase (**Figures 2E–H**).

To gain more insights into the resilience of gut microbial communities, we divided the fecal samples from VTs into two groups according to their enterotypes in Beijing and explored the dynamic changes in these four genera during the long stay. We found that although changes in *Prevotella* and *Bacteroides* differed between enterotype 1 and enterotype 2, the dynamic changes observed in Ruminococcaceae_unclassified and *Bifidobacterium* were similar (**Figure 2I**). Furthermore, we found correlations among the four genera (**Figure 2J**), such as between *Sutterella* and *Bacteroides* (rho = 0.516, *P* = 3.34 × 10^−14^) and between *Clostridium* and Ruminococcaceae_unclassified (rho = 0.676, *P* = 1.88 × 10^−26^).

We further investigated the bi-directional plasticity of the gut microbial communities from the aspect of the genera in the ecological network to gain insights into the plasticity. A total of 109 most abundant genera were clustered into 10 co-abundance groups (CAGs) (**Supplementary Figures S2A, B**). These CAGs were annotated according to the dominant genera, including *Prevotella*, *Bacteroides*, Ruminococcaceae_unclassified, Lanchnospiraceae_unclassified, Bacillaceae_unclassified, *Bifidobacterium*, and Enterobacteriaceae_unclassified. The Wiggum plot (6) showed the co-abundance association networks of genera for each phase and each group and revealed unique patterns of abundances for these 10 CAGs from T1 to T6 phases (**Supplementary Figure S3**). Specifically, during the T1 to T6 phases, the relative abundances of six CAGs showed profound changes, including *Prevotella*, *Bacteroides*, Ruminococcaceae_unclassified, Lanchnospiraceae_unclassified, Bacillaceae_unclassified, and Enterobacteriaceae_unclassified CAGs. The relative abundances of *Prevotella* and *Bacteroides* CAGs were suppressed when the VT members stayed in TAT and reverted after returning to Beijing. In contrast, the relative abundances of Ruminococcaceae_unclassified, Lanchnospiraceae_unclassified, Bacillaceae_unclassified, and Enterobacteriaceae_unclassified CAGs showed a reversed pattern, i.e., the relative abundances were amplified first and then suppressed. To quantitatively measure the plasticity of gut microbial communities, we designed an index, namely the adaptation index, which was calculated by comparing communities of volunteer team members with that of the TTN (**Supplementary Figure S3**). The adaptation index first decreased and then increased. More importantly, the index could serve to better understand the response time of gut microbial communities to alternations of environments. When we monitored the differences of adaptation indices between two neighboring phases, we observed that the adaptation index showed a sharp decrease from T1 to T2 (1.18 × 10^−3^) and a sharp increase from T4 to T5 (0.88 × 10^−3^), with these differences being significantly higher than the difference between T1 and T5 (0.50 × 10^−3^). This indicates a quick response time (within one month) and also a quick recovery time (within one month).

These findings suggested that the bidirectional plastic pattern of the human gut microbial communities was largely driven by the bidirectional quantitative alterations in CAGs. Moreover, the changes in the relative abundances of the genera were the underlying reasons for the bidirectional resilience of enterotypes.

### Microbial Drivers at the Species Level for the Enterotype-Dependent Resilience of Human Microbial Communities

**Figure 3:**
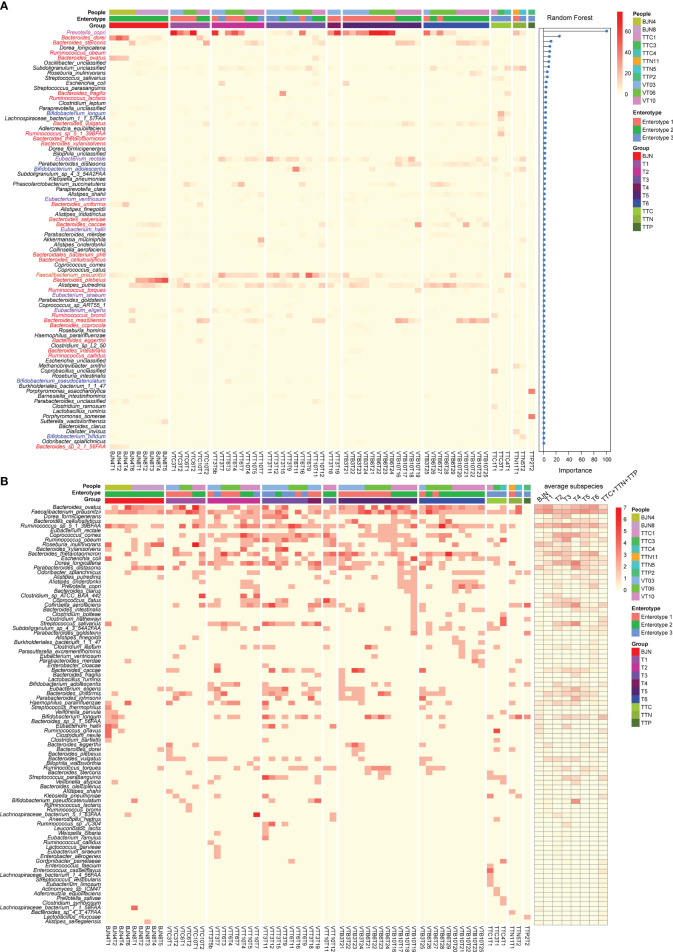
Linking dynamic changes in species and subspecies to the bidirectional resilience of the gut microbial community. **(A)** Dynamic changes in the taxonomic composition of gut microbial communities at the species level. The order of species was sorted by the importance of random forests for identifying the enterotype. **(B)** Dynamic changes in the number of subspecies (left panel) and the average number of subspecies (right panel) during the time series.

To examine differences at the species level and functional differences in the enterotype-dependent resilience of human microbial communities, we selected 62 representative fecal samples for whole-metagenomic sequencing. These samples consisted of time series data for three members of the VT (VT3, VT6, and VT10; 47 samples), two BJNs (9 samples), and three TTNs (3 samples; **Supplementary Figure S1** and **Supplementary Table 1**). These samples can well represent most conditions of each group. For instance, VT3, VT6, and VT10, present non-symptom, abdominal distension, and a condition of an early return, respectively, in a half-year travel. The other selected individuals including BJNs and TTNs were used for comparisons with the VTs. Two BJNs were randomly selected, and three TTNs were selected from three different families. We found that the gut microbial communities in BJNs and VT members at phage T1 who belonged to enterotype 2 varied in *Bacteroides* strain profiles, including *Bacteroides dorei*, *Bacteroides ovatus*, *Bacteroides plebeius*, and *Bacteroides massiliensis*, whereas those with *Prevotella* enriched in enterotype 1 consisted of only a single strain of *Prevotella copri* (**Figure 3A**).

In contrast to the enriched genera profiled by 16S amplicon analysis, we found that the dominant genera in enterotype 3 were *Faecalibacterium*, *Eubacterium*, *Ruminococcus*, and *Bifidobacterium* (**Figure 3A**, **Supplementary Table 2**). The dominant species in enterotype 3 were *Faecalibacterium prausnitzii* and *Eubacterium rectale* (**Figure 3A**, **Supplementary Table 3**). During the long stay, we found that the relative abundances of *Bacteroides dorei*, *Bacteroides plebeius*, and *Prevotella copri* decreased first when the VT members stayed in TAT and then increased after their return. Conversely, the relative abundances of *Faecalibacterium prausnitzii* and *Eubacterium rectale* increased first and then decreased (**Figure 3A**). The transformation of enterotype was caused by changes in these species.

Next, we identified the subspecies in the metagenomic samples and found a series of species with varying subspecies during the study. Importantly, we observed that the average number of subspecies varied considerably when the VT members changed their spatial position (**Figure 3B**). For example, the average number of subspecies of *Bacteroides ovatus* and *Faecalibacterium prausnitzii* immediately decreased from 2.66 and 3.0 subspecies/sample to 1.25 and 2.0 subspecies/sample, respectively when VT members arrived at TAT and increased from 2.0 and 2.5 subspecies/sample to 2.42 and 3.0 subspecies/sample, respectively, after their return (**Figure 3B**, **Supplementary Table 4**). In contrast, *Dorea formicigenerans*, *Ruminococcus* sp. S_1_39BFAA, *Coprococcus comes*, *Dorea longicatena*, and *Streptococcus salivarius* showed an opposite trend (**Figure 3B**, **Supplementary Table 4**). These results suggested that the bidirectional resilience of gut microbial communities was associated with species composition, particularly for the dominant species and their subspecies.

### Functional Plasticity in the Gut Microbial Communities

**Figure 4:**
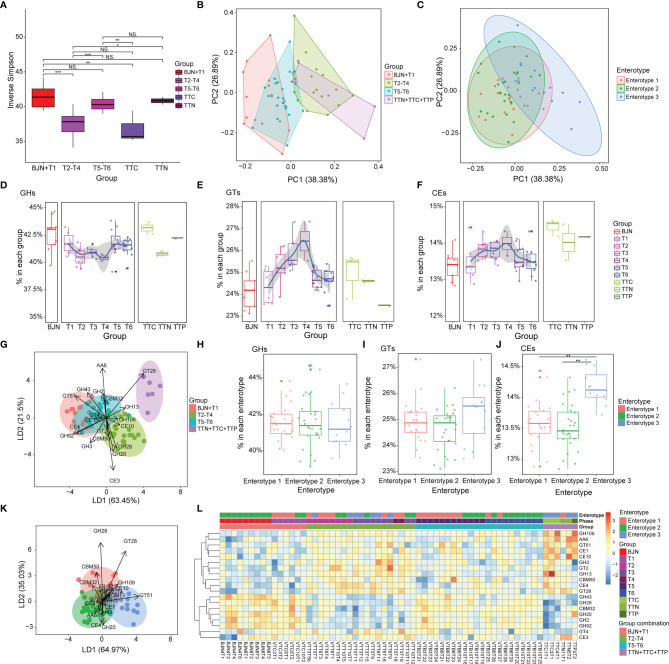
Compositions of carbohydrate metabolism pathways in the human gut microbiome were resilient during the long stay. **(A)** Inverse Simpson index for CAZyme components for microbiome samples from the individuals selected from the cohort of Chinese VT members, TTNs, TTCs, and TTPs. Statistical significance is tested using Wilcoxon test, ***P < 0.001, **P < 0.01, *P < 0.05, and NS, not significant. **(B)** PCA results showing the trajectory of samples, which were colored according to the predefined groups (four groups: BJN-T1, T2–T4, T5–T6, TTN+TTC+TTP), based on the CAZyme component. **(C)** In total, 62 samples were projected to the two-dimensional plane by PCA based on their CAZyme components, and each was then color-labeled and grouped according to their respective enterotype. **(D–F)** Dynamics of the average relative abundance of GHs, GTs, and CEs across the time series. **(G)** Linear discriminant analysis was used to utilize a linear combination of CAZyme components to maximize the separation of the groups. The ellipses represent different groups, and the lengths and directions of the arrows show the normalized scales for each component scaling (for 19 components of CAZyme). **(H–J)** Differences in the average relative abundances of GHs, GTs, and CEs among the three enterotypes. **(K)** Linear discriminant analysis was used to utilize a linear combination of CAZyme components to maximize the separation of the enterotypes. The ellipses represent different groups, and the lengths and directions of the arrows show the normalized scales for each component scaling (for 19 components of CAZyme). **(L)** Heatmap showing the clustering results for CAZyme features and the discernibility of how these CAZyme features differed among groups.

Based on the aforementioned findings, we hypothesized that the bidirectional resilience of the gut microbial community may be associated with dynamic functional changes. To test this hypothesis, we first annotated the predicted genes using the CAZyme database and then divided fecal samples of BJNs and VT members into three groups- BJN-T1 (fecal samples collected in Beijing before departure), T2–T4 (fecal samples collected in TAT), and T5–T6 (fecal samples collected after returning to Beijing). By comparing inverse Simpson indices, we found that the diversity of enzymes for utilizing carbohydrates was significantly different between samples in TAT and samples collected in Beijing (*p* < 0.05, Wilcoxon test, **Figure 4A**). Similarly, by principal component analysis (PCA) of CAZyme profiles generated from microbiome samples, we found that the functional composition of the gut microbial community—which shifted from those similar to BJNs to those similar to TTNs and then reverted after the return—was resilient during the long stay (**Figure 4B**). Additionally, the similarities of functional compositions among these samples were also consistent with the enterotypes (PERMANOVA test based on Euclidean distances with 9,999 permutations: *P* = 0.0897 for enterotype 1 versus 2, *p* < 0.0005 for enterotype 1 versus 3, *p* < 0.0005 for enterotype 2 versus 3) and followed a bidirectional plastic pattern (**Figure 4C**).

### Possible Functional Drivers of the Plasticity of the Human Microbial Communities

We observed that the abundances of glycoside hydrolases (GHs) decreased during the T2–T4 and then increased after the VT member returned to Beijing (**Figure 4D**). In contrast, glycosyltransferases (GTs) and carbohydrate esterases (CEs) increased during phases T2–T4 and then decreased after the VT members returned (**Figures 4E, F**).

We extended our analysis to distinguish groups by a supervised learning approach that integrated a linear combination of the top 19 components of CAZyme (**Figure 4G**). The combination of these 19 components of CAZyme showed considerable power to distinguish fecal samples of VT members collected in Beijing from those of VT members collected in TAT (PERMANOVA, Bray-Curtis distance, permutation = 9,999, *p* < 0.001) and to distinguish fecal samples of VT members collected in Beijing with those from TTNs, TTCs, and TTPs (PERMANOVA, Bray-Curtis distance, permutation = 9,999, *p* < 0.001). However, this combination failed to clearly distinguish T1 samples from T5–T6 samples (PERMANOVA, Bray-Curtis distance, permutation = 9,999, *p* > 0.05; **Figure 4G**).

In addition, no significant differences were observed in the compositions of GHs (**Figure 4H**) and GTs (**Figure 4I**) among three enterotypes, whereas CEs showed significant differences between enterotypes 1 and 3 (*t*-test, *P* = 2.5 × 10^−5^) and between enterotypes 2 and 3 (*t*-test, *P* = 1.95 × 10^−6^; **Figure 4J**).

Similarly, the combinations of these 19 components of CAZymes exhibited considerable power for distinguishing fecal samples of enterotype 1 from those of enterotype 3 (PERMANOVA, Bray-Curtis distance, permutation = 9,999, *P* = 0.001; **Figure 4K**) and those of enterotype 2 from those of enterotype 3 (PERMANOVA, Bray-Curtis distance, permutation = 9,999, *P* = 0.001; **Figure 4L**). These representative enterotype-dependent guilds (40) highlighted the resilience pattern.

Based on these dynamic patterns observed for GHs, GTs, and CEs during the long stay and for different enterotypes, we speculated that the bidirectional resilience of the microbial community may be associated with the metabolism of carbohydrates from different diets.

**Figure 5:**
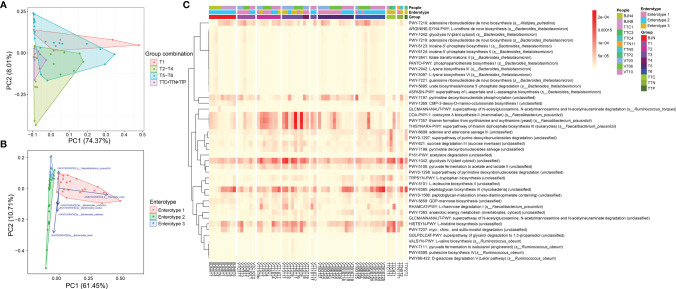
Functional pathways contributed to the bidirectional resilience of human gut microbial communities. **(A)** PCA results showing the trajectory of samples, which were colored according to their defined groups (four groups: T1, T2– T4, T5– T6, TTN+ TTC+ TTP), based on the component of stratified functional pathways. **(B)** PCA results showing the trajectory of samples, which were colored according to the three enterotypes, based on the component of stratified functional pathways. **(C)** Heatmap showing the compositions of enriched and significant functional pathways, which contributed to the bidirectional resilience of the human gut microbial community, across all metagenomics samples.

Accordingly, we performed the PCAs to trace the dynamics of metabolic pathways according to the different groups (**Figure 5A**) and three enterotypes (**Figure 5B**). We found that the metabolic compositions of the VT members during T1 differed slightly from that of samples in T5–T6 phase (PERMANOVA, Bray-Curtis distance, permutation = 9,999, *P* = 0.4639). In contrast, there was a significant difference between the metabolic composition of the VT members at T1 and that of samples at T2–T4 (PERMANOVA, Bray-Curtis distance, permutation = 9,999, *P* = 0.0054; **Figure 5A**). Moreover, we found that there were significant differences among the compositions of the three enterotypes (PERMANOVA, Bray-Curtis distance, permutation = 9,999, *P* < 0.005; **Figure 5B**). Among the functional pathways, we observed that unintegrated pathways of *Prevotella copri*, *Bacteroides* sp. (e.g., *Bacteroides dorei* and *Bacteroides plebeius*), and *Faecalibacterium prausnitzii* corresponded to enterotypes 1, 2, and 3, respectively (**Figure 5B**). For other known pathways, we found dynamic changes in coenzyme A biosynthesis II, thiamin formation from pyrithiamine and oxythiamine, and glucose metabolism, including glycolysis IV and pyruvate fermentation to acetate and lactate II, also exhibited a plastic pattern (**Figure 5C**). The relative abundances of these pathways increased when VT members arrived at TAT, remained stable during the stay at TAT, and decreased after return to Beijing.

### Dietary Shifts Were Correlated With the Plastic Pattern of the Human Microbial Communities

**Figure 6:**
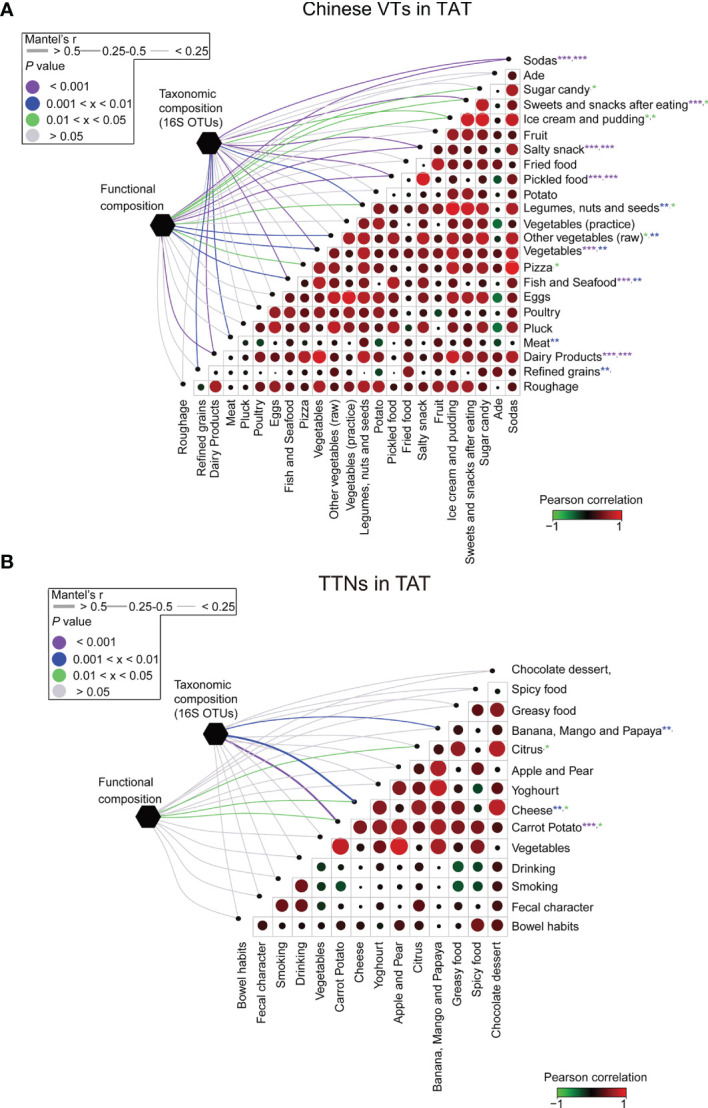
Association between diet and the structural and functional compositions of the microbial communities for VT members and TAT natives. The associations between dietary variables and the taxonomic and functional compositions for **(A)** VT members staying in TAT and **(B)** TAT natives. Circle sizes in the matrices represent the absolute values of Pearson correlation coefficients for the two corresponding factors, whereas circle colors represent either positive or negative correlations. The edge widths represent the Mantel’s *r* statistic for distance correlations, and the edge color denotes the statistical significance based on 9,999 permutations. ***P < 0.001, **P < 0.01, and *P < 0.05.

The VT members exhibited dietary habits considerably different from those in Beijing during their stay in TAT. Among their diets, the increased consumption of foods such as fish, seafood, dairy products, and refined grains was significantly correlated with changes in gut microbial community structures (*p* < 0.05, **Figure 6A**). However, in contrast to VT members, the composition of gut microbial communities in TAT natives was tightly associated with the consumption of bananas, mangos, papayas, cheese, and carrot potatoes (*p* < 0.05, **Figure 6B**). These findings are consistent with previous studies demonstrating that changes in diet affect the composition of the human gut microbial communities (7, 41–43). Therefore, the diet in TAT, which was different from that in Beijing, may have led to changes in the gut microbial communities of VT members during this study.

## Discussion

This longitudinal study was based on high-density sampling (average of more than 20 per individual) of volunteers with multiple dietary shifts, and reported the dynamic variation of the human gut microbiome in a whole half-year travel, which was reflected by the switching and restoring of enterotypes.

The enterotypes switch and alterations in specific driven species might exert effects on human immunity. We found that the resilience of taxonomical and functional compositions of the human gut microbiome was highly enterotype-specific. The identified genera and species that may drive the observed plasticity included variations in *Prevotella*, *Bacteroides*, Ruminococcaceae_unclassified, and *Bifidobacterium* at the genus level and *Bacteroides dorei*, *Bacteroides ovatus*, *Bacteroides plebeius*, *Bacteroides massiliensis*, *Prevotella copri*, *Faecalibacterium prausnitzii*, and *Eubacterium rectale* at the species level. Many of these microbial species were reported to be correlated with immunity modulation. For instance, *Bacteroides dorei* was reported to be depleted in patients with coronary artery disease (CAD), and the gavage of *B. dorei* could attenuate atherosclerotic lesion formation in atherosclerosis-prone mice, notably ameliorating endotoxemia followed by decreasing gut microbial lipopolysaccharide production, effectively suppressing pro-inflammatory immune responses (44). In addition, *B. ovatus* was reported to produce indole-3-acetic acid that promoted IL-22 production by immune cells, yielding beneficial effects on colitis (45). Moreover, *P. copri* was reported to play a crucial role in rheumatoid arthritis (RA) pathogenesis, a chronic autoimmune disease (46). *P. copri* were found expanded in patients with new-onset RA (47), and the HLA-DR-presented peptide identified from the 27-kDa protein of *P. copri* was capable of stimulating a TH1 cell response in 42% of patients with new-onset RA (48). Other species have also been linked to immune diseases, such as *B. massiliensis* related to the severity of COVID-19 (49), and *E. rectale* related to the pathogenesis of ulcerative colitis and inflammatory bowel disease (50). Therefore, if people traveled to a place where dominated enterotypes differed from theirs, the change in environment such as diet would render it possible for people to switch their enterotype, bringing about the potential risk of immunity disorders.

From a functional perspective, the bidirectional resilience of gut microbial communities was primarily driven by a few functional groups (guilds) related to carbohydrate metabolism, e.g., GHs, GTs, and CEs, and a series of glucose metabolic pathways. We found GHs were decreased but GTs and CEs increased during the travel, and these functions were then restored after VTs returned to Beijing. Carbohydrate metabolism can modulate innate immunity (51), thus the change in the ability of carbohydrate metabolism during the travel, together with the taxonomic composition, warranted a microbial intervention to maintain health.

The taxonomic and functional changes in the gut microbiome were found mediated by dietary change. For instance, VT people had increased consumption of seafood, dairy products, and refined grains during their stay in TAT, which were significantly correlated with their microbiome variation. Accordingly, prior prediction of one’s microbial resilience patterns could facilitate the preparation of travelers. Proper dietary guidance, designed according to an individual’s original enterotype and the dominated enterotype in the destination, would be beneficial for the individual to maintain the enterotype stability in travel for months.

More efforts are needed to improve our understanding of the plastic patterns of the human gut microbiome after a half-year travel and their health implications. First, many confounding factors, including living conditions and disruption of the circadian rhythm, may influence the dynamics of the human gut microbiome, however, the mechanisms remained unknown. Second, the effects of genetic factors in shaping the enterotype-dependent plastic patterns of the gut microbiome should be examined. Third, the relationships between enterotype-dependent plastic patterns in the gut microbiome and the health status of the host needs further experiments and detailed clinical records. These issues should be addressed in future studies.

Collectively, this study has profiled the longitudinal dynamics of human gut microbial communities in a travel for months, and opened new avenues for probing the effects of diet and environments on human gut microbial communities, as well as the implications of human gut microbial communities on immunity and other health indicators.

## Data Availability Statement

All sequencing data (including 287 16S rRNA data and 62 metagenomic data) for fecal samples were deposited in NCBI’s Sequence Read Archive database under Bioproject number PRJNA393237.

## Ethics Statement

Ethical review and approval was not required for the study on human participants in accordance with the local legislation and institutional requirements. The patients/participants provided their written informed consent to participate in this study.

## Author Contributions

This study was designed by MC, HL, MH, JW, GT, and KN. MC, HL, MH, ZH, RW, YL, FC, JP, HP, HS, YX, LC, QZ, and FG collected samples. MC, HL, MH, RW, and PY analyzed the data. MC, HL, MH, SL, DB, SS, and KN wrote the initial draft of the manuscript. All authors revised the manuscript. This study was supervised by JW, GT, and KN. All authors contributed to the article and approved the submitted version.

## Funding

This work was partially supported by National Natural Science Foundation of China (grant nos. 31871334, 32071465, 31671374, 31327901, 31671369, 31770775, 61521092, 91430218, 61472395, and 61432018), National Key Research and Development Program of China (grant nos. 2018YFC0910502 and 2018YFC0910405), and the GRF Research Project 9042348 (grant no. CityU11257316).

## Conflict of Interest

The authors declare that the research was conducted in the absence of any commercial or financial relationships that could be construed as a potential conflict of interest.

## Publisher’s Note

All claims expressed in this article are solely those of the authors and do not necessarily represent those of their affiliated organizations, or those of the publisher, the editors and the reviewers. Any product that may be evaluated in this article, or claim that may be made by its manufacturer, is not guaranteed or endorsed by the publisher.
