# Supplementary material for: Microbiome Resilience and Health Implications for People in Half-Year Travel
Source: Front Immunol. 2022 Feb 24;13:848994. doi: 10.3389/fimmu.2022.848994 (PMC8907539; doi:10.3389/fimmu.2022.848994)
Supplement: Supplementary file 1 [file DataSheet_1.pdf]

Supplementary Material

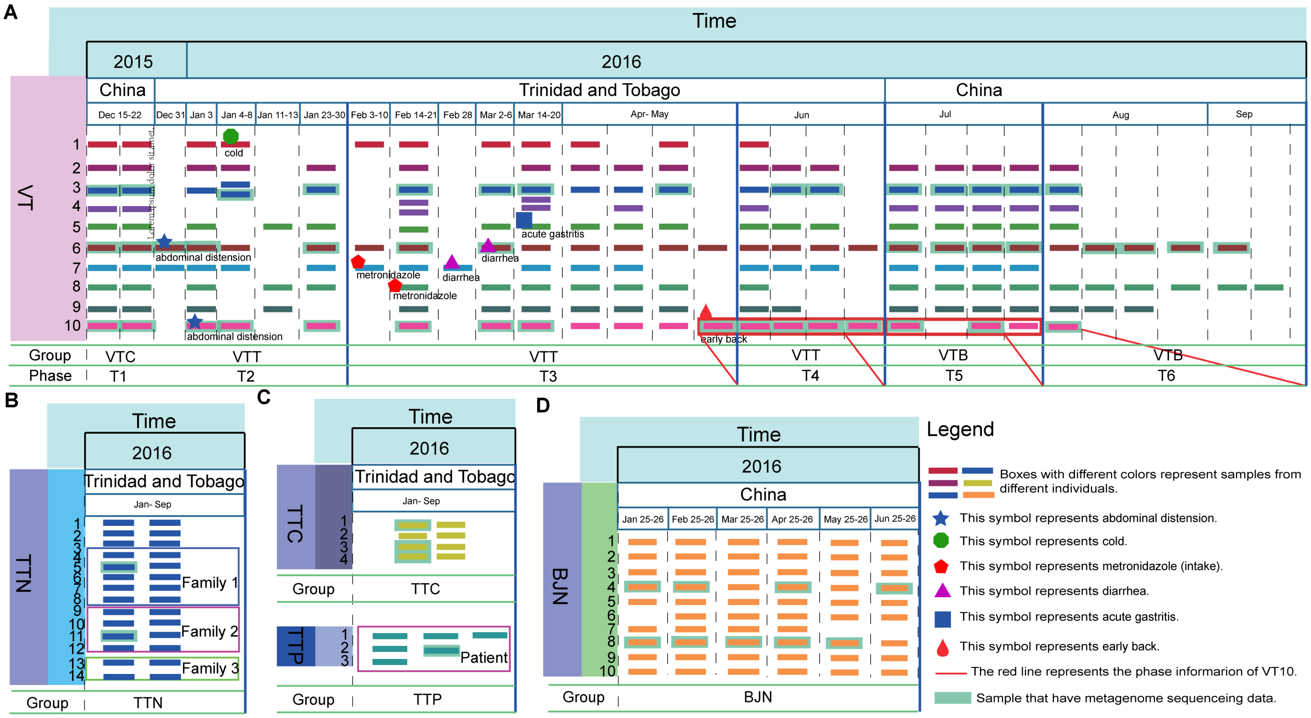

Supplementary Figure S1 | Sample, group, phase, and sequencing information. Faecal sample information of **(A)** Chinese volunteer team (from T1 to T6), **(B)** Trinidad and Tobago native (TTN), **(C)** Trinidad and Tobago Chinese (TTC, who stayed in TAT for more than one year) and Trinidad and Tobago patient (TTP), **(D)** Beijing native (BJN). Based on the information of time-series, we divided faecal samples of volunteer team into 6 phases, including T1, T2, T3, T4, T5, and T6, in which T1 represents pre-travel phase time-slot, T2, T3 and T4 represent 3 time-slots when the volunteer team stayed in TAT, and T5 and T6 represent 2 time-slots after the volunteer team return to Beijing, China. In this figure, boxes with different colors represent different faecal samples from different individuals. Special events are also annotated, such as catch a cold and take medicines. Boxes with shadings indicated samples with metagenome sequenced data. The red line and red box represent special phase information for VT10 (early return to Beijing).

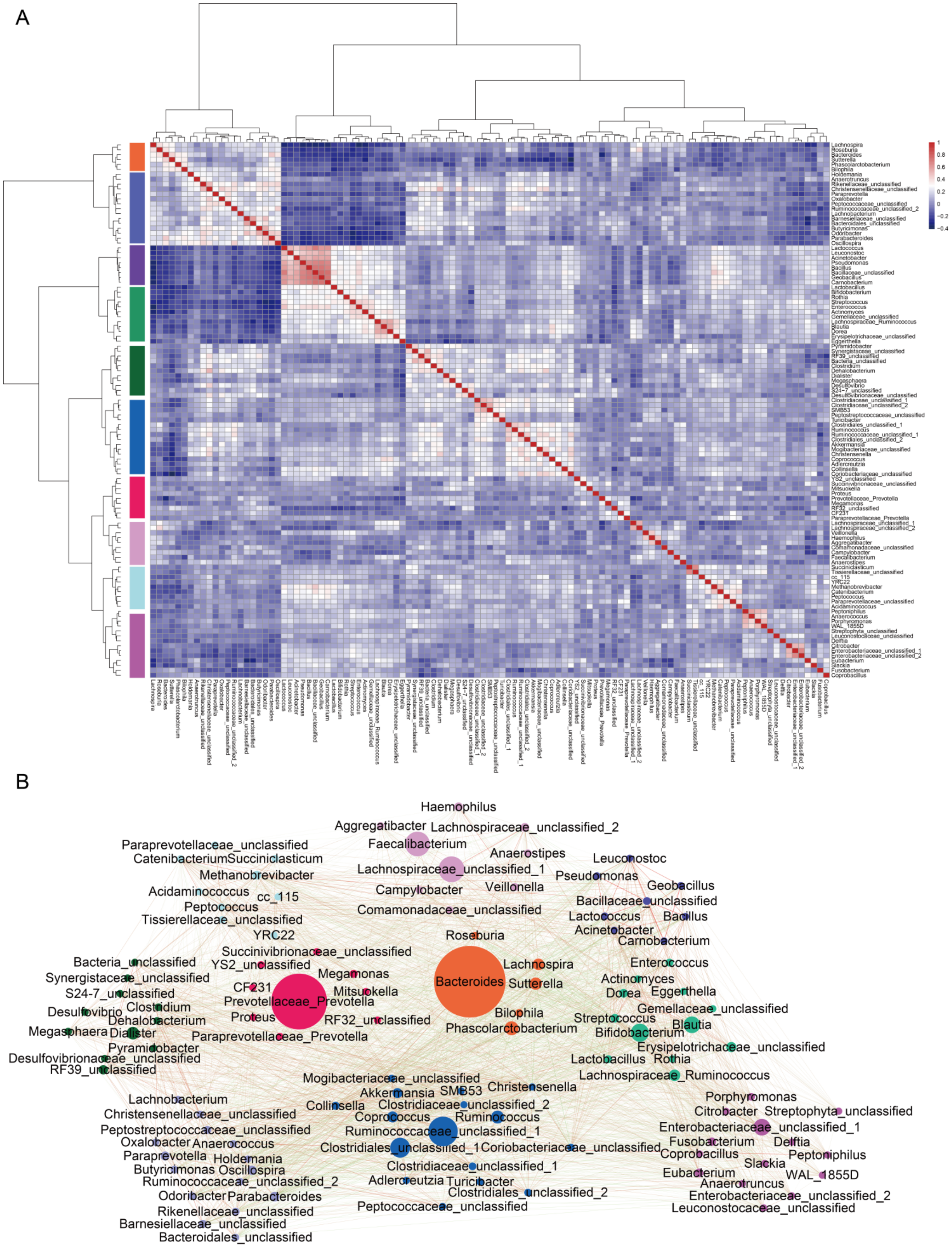
A
B
Haemophilus
Aggregatibacter
Lachnospiraceae_unclassified_2
Paraprevotellaceae_unclassified
Faecalibacterium
Anaerostipes
CatenibacteriumSucciniclasticum
Leuconostoc
Methanobrevibacter
Lachnospiraceae_unclassified_1
Pseudomonas
Acidaminococcus
Campylobacter
Veillonella
Geobacillus
cc_115
Peptococcus
Comamonadaceae_unclassified
Bacillaceae_unclassified
Tissierellaceae_unclassified
Lactococcus
Bacillus
Acinetobacter
YRC22
Roseburia
Carnobacterium
Succinivibrionaceae_unclassified
Bacteria_unclassified
YS2_unclassified
Lachnospira
Enterococcus
Megamonas
Synergistaceae_unclassified
Actinomyces
CF231
Bacteroides
Sutterella
S24-7_unclassified
Mitsuokella
Dorea
Eggerthella
Prevotellaceae_Prevotella
Clostridium
Desulfovibrio
Gemellaceae_unclassified
Bilophila
Dehalobacterium
Proteus
RF32_unclassified
Streptococcus
Blautia
Megasphaera
Dialister
Phascolarctobacterium
Bifidobacterium
Paraprevotellaceae_Prevotella
Pyramidobacter
Erysipelotrichaceae_unclassified
Desulfovibrionaceae_unclassified
Lactobacillus
Rothia
RF39_unclassified
Mogibacteriaceae_unclassified
Lachnospiraceae_Ruminococcus
Christensenella
Akkermansia
SMB53
Porphyromonas
Lachnobacterium
Collinsella
Clostridiaceae_unclassified_2
Citrobacter
Streptophyta_unclassified
Christensenellaceae_unclassified
Coprococcus
Ruminococcus
Peptostreptococcaceae_unclassified
Ruminococcaceae_unclassified_1
Enterobacteriaceae_unclassified_1
Oxalobacter
Anaerococcus
Fusobacterium
Delftia
Clostridiales_unclassified_1
Coriobacteriaceae_unclassified
Paraprevotella
Holdemania
Coprobacillus
Peptoniphilus
Slackia
Butyricimonas
Oscillospira
Clostridiaceae_unclassified_1
Ruminococcaceae_unclassified_2
Eubacterium
WAL_1855D
Adlercreutzia
Turicibacter
Anaerotruncus
Odoribacter
Parabacteroides
Clostridiales_unclassified_2
Enterobacteriaceae_unclassified_2
Rikenellaceae_unclassified
Peptococcaceae_unclassified
Leuconostocaceae_unclassified
Barnesiellaceae_unclassified
Bacteroidales_unclassified

Supplementary Figure S2 | Evidence behind the categorization of microbial Co-abundance groups (CAGs). CAGs were categorized based on the information shown in **(A)** heatmap showing Kendall correlations between genera clustered by the Spearman correlation coefficient and Ward linkage hierarchical clustering. The clustering tree was divided into 10 clusters (CAGs), and the detailed information was shown in **(B)** Network plot for the whole cohort studied. Circle sizes indicated the average abundances of the genus. The color of the edge represents the negative or positive correlation between genus (nodes). The thickness of the lines (edges) is proportional to correlation strength.

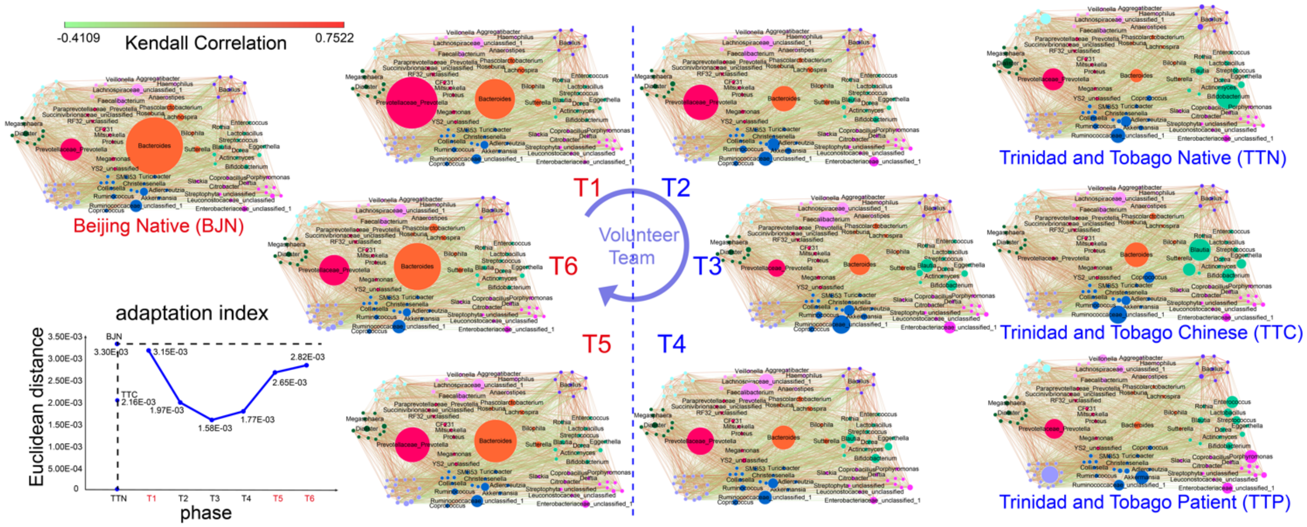

Supplementary Figure S3 | Networks indicate quantitative commons and differences among ten CAGs in groups/phases. Each node represents a microbial genus and its area is proportional to the mean relative abundance within the group/phase. Connections between nodes represent significant Kendall correlation between genera (FDR<0.05). The red line and green line represent positive correlation and negative correlation, respectively. The adaptation index quantifies the bi-directional plasticity. The adaptation indices for TTC and BJN samples have been computed and shown in dashed lines as references.
